# Information bubble and skill evolution: a theoretical framework for integrating distributed cognition, cognition offloading, and metacognitive regulation based on AIOE

**DOI:** 10.3389/frai.2026.1768178

**Published:** 2026-07-29

**Authors:** Tansheng Lu, Zihan Cheng

**Affiliations:** 1 School of Business, Tongda College of Nanjing University of Posts and Telecommunications, Yangzhou, Jiangsu, China; 2 School of Sociology and Population Studies, School of Social Work, Nanjing University of Posts and Telecommunications, Nanjing, Jiangsu, China

**Keywords:** artificial intelligence occupation exposure, cognitive offloading, distributed cognition, human-computer complementarity, information bubble, metacognitive regulation, skill evolution

## Introduction

With the exponential growth of Artificial Intelligence (AI) technology, especially the widespread application of Generative AI (GenAI), AI is no longer just a passive tool to assist human work, but has transformed into an environmental factor that reshapes work paradigms and defines the boundaries of professional skills. To quantify the depth and breadth of this technological penetration, scholars have introduced the core concept of “Artificial Intelligence Occupational Exposure” (AIOE). In this theoretical framework, Artificial Intelligence Occupational Exposure is defined as the extent to which the specific tasks and expertise requirements of an occupation overlap with the operational capabilities of generative AI systems. This concept is increasingly characterized by two distinct functional dimensions, namely automation, which describes the substitution of routine cognitive operations, and augmentation, which involves the synergy between human expertise and AI in high-order analytical tasks. Consequently, individuals in high-exposure roles experience significant cognitive restructuring rather than simple labor displacement, a distinction supported by the latest research of MacGill (2018). Such exposure creates a dynamic environment for skill evolution, where the outcome depends on the individual's ability to navigate technological affordances, as evidenced by the large-scale analysis of Zentz (2024). The pioneering research of Felten et al. (2021) shows that AIOE is not uniformly distributed but constitutes a complex hierarchy of professional contexts: from deep exposure faced by high-cognitive-load professions such as financial auditors and genetic counselors, to relatively low exposure faced by physical laborers, this difference constitutes a fundamental feature of the modern labor market. However, shifting focus from macrolevel professional structures to microlevel individual psychological adaptation reveals a paradox: identical AIOE environments do not yield uniform adaptive outcomes. Instead, they precipitate significant differentiation in individual cognitive patterns and skill structures.

This divergence manifests in two distinct psychological and behavioral trajectories, igniting robust debate within the academic community. On one hand, pessimistic perspectives warn that high-intensity AIOE may precipitate “AI-induced cognitive atrophy” (AICICA). Dergaa et al. (2024) posit that, analogous to muscular atrophy from disuse, overreliance on AI chatbots may induce the degeneration of critical thinking, memory encoding, and decision-making faculties. This state is termed a “cognitive echo chamber”—a pathological condition characterized by algorithmic entrapment, narrowed information intake, and diminished cognitive processing capabilities. Conversely, empirical evidence from labor economics offers an optimistic counter-narrative: the potential for skill evolution. Engberg et al. (2025), analyzing Swedish employment data, found that firms with high AI exposure exhibited increased recruitment, underscoring the complementarity between AI and labor. Similarly, Baek and Lee's (2025) comparative study reveals that in the United States, AI exposure drives growth in both wages and employment.

Why does the same AIOE environment lead to such polarized results? The existing technological determinism perspective is difficult to explain the differences in this micro-mechanism. Recent empirical studies corroborate this paradox. On the negative adaptation spectrum, research on cognitive offloading indicates that excessive reliance on AI systems can short-circuit the cognitive effort required for deep learning, creating a state of “false mastery” that leads to cognitive atrophy (Lodge and Loble, 2026). Conversely, the proactive adaptation path is supported by Zhong et al. (2026), whose empirical findings reveal a nonlinear, U-shaped relationship between AI-induced job insecurity and innovative behavior. This suggests that high AIOE can actually catalyze innovative work behavior when individuals possess high creative self-efficacy and adopt problem-focused coping mechanisms. Green et al. (2025) found that the subjective expectations of workers regarding the impact of AI (“winners” and “losers”) are not entirely consistent with the objective exposure, which suggests that we must pay attention to the internal psychological regulatory mechanisms of individuals. This paper aims to fill this theoretical gap and proposes the following core hypothesis: the AIOE environment reshapes individual cognitive load through the cognitive offloading mechanism, and metacognitive regulation is the key differentiated variable that determines whether this offloading leads to a cognitive bubble or skill evolution. We will construct a theoretical framework integrating distributed cognition, cognitive offloading, and metacognitive regulation to explain this process.

## AI as a cognitive environment: reconstruction of human-machine relationships in distributed cognitive systems

To understand the profound impact of AIOE on individuals, it is first necessary to break away from the traditional perspective of viewing AI merely as a tool and to re-conceptualize it as a cognitive environment. The extended mind theory proposed by Clark and Chalmers (1998) suggests that cognitive processes are not confined to the brain but extend to the external environment. In the AIOE environment, this extension reaches a new height, forming a tightly integrated “human-machine” distributed cognitive system. Yang et al. (2025) argue that generative AI expands cognitive construction from the isolated individual mind to a distributed sociotechnical system. This transformation not only reconstructs professional cognitive boundaries but also fundamentally alters the logic of knowledge production within this intelligent enhancement environment. Here, AI transcends its role as a mere information repository to become an active cognitive partner, engaging in simulated dialogue and adaptive interaction.

This partnership manifests a significant duality across diverse professional domains. On the affirmative side, by assuming computationally intensive tasks, AI serves as a potent extension of human cognition. For instance, in radiation protection, Fazli et al. (2025) demonstrated that deep learning-driven real-time monitoring significantly enhances safety efficiency. Similarly, in nursing, Zhou and Fang (2022) reported an 87% concordance between AI-assisted systems and diagnoses by experienced practitioners, effectively alleviating cognitive load and enabling a shift toward higher-value care. Conversely, such deep integration introduces inherent risks. Dergaa et al. (2024) caution that the anthropomorphic nature and immediate feedback of AI chatbots may foster excessive trust, shifting the user dynamic from tool utilization to partner dependency. Furthermore, Cui and Wei (2026) emphasize that during multimodal data processing, algorithmic “black boxes” and semantic gaps may exacerbate this trust crisis, creating an accountability vacuum. Consequently, AIOE functions as a tension-laden cognitive ecosystem, offering resources for enhancement while simultaneously exerting pressure toward cognitive inertia.

## The generation mechanism of cognitive echo chambers: non-adaptive cognitive offloading and algorithmic dependence

Cognitive echo chamber is a negative psychological outcome that individuals may face in the AIOE environment, its essence being the degradation of cognitive ability and the narrowing of perspective. Its formation mechanism mainly originates from a maladaptive cognitive offloading pattern. Firstly, from the perspective of cognitive offloading, individuals may adopt passive or opportunist cognitive offloading strategies. The research of Wei et al. (2025) points out that generative AI tools such as ChatGPT greatly accelerate the process of cognitive offloading. When individuals are driven by the principle of effort reduction and indiscriminately outsource high-level cognitive tasks such as logical reasoning and critical thinking to AI, although it releases short-term cognitive resources, it also leads to the atrophy of core cognitive functions due to the lack of necessary difficulty training, that is, the effect of “use it or lose it”. That is, the effect of “use it or lose it.” This “uncalibrated offloading” fundamentally undermines what Sidra and Mason (2026) describe as “cognitive sovereignty.” When individuals fail to maintain metacognitive oversight during AI interaction, the distributed cognitive system shifts from a collaborative partnership to a state of partner dependency, where the human element becomes a passive consumer of algorithmic outputs rather than an active controller.

Secondly, intensifying algorithmic dependence has exacerbated the entrenchment of information cocoons. Wu and Wen (2025) indicate that within opaque contexts or specific incentive structures, individuals are prone to developing blind “algorithmic appreciation.” This tendency allows algorithmic recommendation logic to dominate information intake pathways, eroding the volition to actively seek diverse perspectives. Wen and Wu (2025) further analyze structural determinants, positing that such dependency is not merely an individual choice but is conditioned by societal “technological halo” effects and an “efficiency-first” culture. As individuals habituate to immediate AI feedback, their capacity for metacognitive monitoring and regulation atrophies. Consequently, they become less adept at detecting algorithmic errors or biases, thereby sliding into a cognitive echo chamber. Xiang's (2025) analysis of the “Generation Z” cognitive landscape corroborates this view, suggesting that insufficient information literacy may cause digital natives to lose subjectivity amidst algorithmic saturation, resulting in cognitive constriction and ossification.

## Skill evolution driving mechanism: human-machine complementary and strategic cognitive resource allocation

In contrast to cognitive echo chambers, skill evolution represents a positive adaptive outcome, where individuals achieve the expansion and upgrade of their abilities through human-computer interaction. This process depends on human-computer complementarity and strategic cognitive offloading. The core of achieving skill evolution lies in the individual's ability to use AI as a cognitive lever. This strategic utilization of AI as a cognitive lever is specifically empowered by two core components of AI literacy, namely technical understanding and critical evaluation. According to the scoping review by Roshanaei (2024), a robust understanding of how AI models process data allows individuals to predict potential errors and identify algorithmic biases. This technical insight transforms cognitive offloading from a passive act of effort reduction into a deliberate strategy of resource re-allocation. Furthermore, as argued by Yan et al. (2025), high-level AI literacy enables users to scrutinize machine-generated content through the lens of critical thinking, which ensures that the human agent remains the primary arbiter of quality and accuracy. By possessing these competencies, individuals can effectively navigate the complexities of AIOE and maintain their cognitive sovereignty within the distributed system. Engberg et al. (2025) found in their study of the Swedish job market that companies with high AI exposure tend to use AI to enhance the productivity of existing positions rather than simply replace labor, which reflects the complementarity of AI. In this process, individuals achieve cognitive reconstruction through strategic offloading—that is, offloading repetitive, low-value tasks while retaining higher-order thinking tasks.

From the perspective of distributed cognition, this is the result of individuals actively managing the “human-machine” system. Wang and Liang (2025) found in their research in the logistics industry that with the improvement of intelligence level, the skill requirements have shifted from single operation to system planning and dynamic optimization. Practitioners are no longer passive executors of the system but have transformed into managers and developers. This skill upgrade has been supported at the macro level by Georgieff and Hyee (2022), who found that high-skilled digital workers can better collaborate with AI, use technology to improve productivity, while low-skilled workers face the risk of replacement. In addition, Lábaj et al. (2025) in their research in the UK, although they found that AI may impact high-skilled jobs, also pointed out the role of technological complementarity in skill reconstruction. For individuals who can adapt to new paradigms, AI has become an “external brain” that expands the cognitive boundaries, promoting a spiral rise in skills. Giuntella et al. (2025) further found in their research in Germany that this adaptability not only brings economic benefits but also improves workers' health and well-being by reducing the physical burden of work.

## Integrated theory model: evolutionary path from environmental input to psychological outcome

To clearly elucidate the cognitive and skill evolution process of individuals in the AIOE environment, we have constructed a theoretical framework that integrates distributed cognition, cognitive offloading, and metacognitive regulation. The logical starting point of this framework is environmental input, that is, AIOE stimuli. These stimuli not only include new experiences brought by AI but also sources of stress such as processing massive information and coping with technological updates. According to the classification by Felten et al. (2021), these inputs manifest as high AIOE (such as knowledge-intensive service industries) and low AIOE (such as labor-intensive industries) differences across different professions.

Environmental input then enters the individual cognitive system, which is the core processing hub of the model. At this stage, the three elements of metacognitive regulation, cognitive strategies, and motivational orientation interact with each other. Metacognitive regulation, as the core variable, determines the level of monitoring and evaluation of environmental input by individuals. The efficacy of this metacognitive regulation is fundamentally contingent upon the individual's level of artificial intelligence literacy. As conceptualized in the systematic review by Rupnik and Avsec (2025), AI literacy represents a multidimensional construct that encompasses the foundational knowledge of algorithmic functions and the ability to critically engage with AI outputs. Within our integrated framework, AI literacy serves as the cognitive infrastructure that supports metacognitive monitoring, providing the necessary technical context for individuals to calibrate their trust in system performance. As described by Lin and Jiang (2025), components such as belief control directly affect individuals' judgments of task value. Cognitive strategies involve whether individuals choose exploratory learning or passive offloading, while motivational orientation (growth-oriented vs. fixed-oriented) provides the psychological motivation for this choice.

Via cognitive processing, individuals generate specific schema adjustments, translating them into concrete behavioral outputs. Schema adjustment entails reconstructing one's knowledge structure, categorized into high-adaptive forms (e.g., adapting to new skill requirements) and low-adaptive states (e.g., skill ossification). These adjustments dictate skill decision-making trajectories—either assimilating AI capabilities or passively accepting substitution—thereby influencing professional outcomes. Drawing on Yu and Zhang (2024), this decision matrix varies across professions; notably, high-exposure roles face a critical need to avert skill ossification.

Ultimately, this process crystallizes into two distinct psychological outcomes: a cognitive echo chamber or skill evolution. The former manifests as algorithmic dependency and cognitive regression, whereas the latter involves human-machine complementarity and the expansion of cognitive boundaries. The model constitutes a closed feedback loop wherein psychological outcomes reinforce or weaken the individual's metacognitive state, generating a cumulative effect over time.

## Key differentiation mechanism: the core hub role of metacognitive regulation

The core insight of this framework lies in the fact that AIOE itself is neutral, metacognitive regulation is the key bridge connecting individual cognitive systems and cognitive schema adjustments, and it is also the fundamental cause determining the differentiation of results. Metacognitive regulation functions as the primary internal evaluator of the extrinsic cognitive load induced by these AI systems. When individuals interact with high-complexity AI outputs, their metacognitive monitoring level determines whether the process remains a critical evaluation or becomes a form of uncalibrated cognitive offloading. Recent empirical evidence from Gerlich (2025) suggests that this process is fundamentally bidirectional. While persistent exposure can increase cognitive convenience, it often imposes significant metacognitive costs by impairing the user's ability to accurately judge system performance, a phenomenon identified by Welter et al. (2022). Therefore, robust metacognitive regulation is required to transform AI from a disruptive influence into a scaffold for cognitive expansion, ensuring that the human agent remains the primary controller within the distributed cognitive system. The research by Lin and Jiang (2025) confirms that in the generative AI environment, students' belief control (a component of metacognition) can significantly predict their judgments of task value and influence learning motivation through the mediating role of self-efficacy. Self-efficacy and goal orientation together constitute the transmission path of “belief-efficacy-goal-value,” deepening the understanding of the motivation system. Specifically, high metacognitive monitoring guides individuals to evaluate the value of AIOE stimuli and choose deep processing modes. Individuals with this ability can perceive the limitations of AI, thereby inhibiting blind conformity, adopting strategic offloading and exploratory learning, and ultimately achieving high adaptive adjustments. Conversely, low metacognitive monitoring leads individuals to blindly rely on the convenience of AI, activating simplified processing modes, and adopting passive offloading, ultimately resulting in low adaptive adjustments. This mechanism explains why individuals facing high AIOE can better adapt, while some groups (such as highly educated males as pointed out by Jetha et al., 2025) can better adapt, while others (such as moderately skilled females) face greater substitution risks. The level of metacognitive regulation essentially determines the power status of individuals in the distributed cognitive system—they become the controller of the system or become the dependent of the system.

## Evolutionary path analysis: from cognitive processing to steady state

Within the AIOE environment, individual adaptation is not a stochastic process; rather, it diverges along two distinct trajectories, culminating in stable psychological and behavioral states. The first trajectory, the passive dependency path, originates from low metacognitive monitoring and a fixed mindset. Here, individuals tend to adopt opportunistic cognitive offloading strategies, outsourcing core cognitive tasks to AI primarily for effort minimization. Consequently, the absence of deep processing causes cognitive schemas to stagnate and ossify through disuse, precipitating a decline in professional competitiveness. This dynamic establishes a vicious cycle that reinforces algorithmic dependency, ultimately entrapping individuals within a “cognitive cocoon” defined by information narrowing and the erosion of subjectivity. Jaccoud's (2025) findings on wage inequality within professions also support this point, that is, the lack of adaptability can lead to wage stagnation.

In sharp contrast is the proactive adaptation path. This path is driven by high-level metacognitive monitoring and growth mindset. Individuals not only view AI as a tool but also as a challenge and scaffold, adopting strategic offloading to retain higher-order thinking training. Driven by high-adaptability adjustments, individuals expand their cognitive boundaries and enhance professional effectiveness by assimilating new skills and accommodating new paradigms. This positive feedback loop continuously strengthens individuals' sense of self-efficacy, ultimately leading to a state of human-computer complementary skill evolution. This proactive adaptation is fundamentally anchored in the exercise of human agency. According to the theoretical framework proposed by Torres-Martínez (2025), human agency serves as the primary driver for achieving a balanced integration between machine intelligence and human intuition. Such agency manifests as the capacity to delegate routine functions while simultaneously reclaiming cognitive resources for complex problem solving. This deliberate allocation of cognitive effort facilitates a complementary rather than competitive relationship with AI systems. Consequently, the active adaptation path represents an agentic evolution where the individual remains the architect of the collaborative workflow. To more clearly demonstrate the essential differences between these two paths in multiple dimensions such as psychological mechanisms, behavioral patterns, and long-term consequences, and to provide a theoretical and empirical comparative analysis, we constructed Table 1.

**Table 1:** Comparative multidimensional features and results of passive dependency and active adaptation paths in the AIOE environment.

Comparison dimensions	Passive dependency path features	Active adaptation path features
Metacognition and motivation regulation state	Individuals exhibit low levels of monitoring ability, lack awareness of cognitive processes, tend to have fixed mindset, and are prone to blind trust in algorithms or irrational feelings of threat.	Individuals maintain a high level of monitoring and regulation, continuously assessing the accuracy and value of AI outputs, possess a growth mindset, and view technology as a scaffold for self-improvement.
Cognitive offloading strategy pattern	Adopting an opportunist offloading mode, driven by the principle of cognitive thrift, tends to outsource cognitive tasks comprehensively for the sake of effort-saving, including core judgment and reasoning.	Adopt a strategic offloading mode, selectively outsourcing low-level repetitive tasks based on the nature of the tasks, and deliberately retaining the necessary difficulty of high-level tasks to train thinking ability.
Skill schema evolution direction	Cognitive schemas become solidified or alienated, existing skills degenerate due to long-term disuse, new adaptive schemas are not established, and a significant “de-skillization” phenomenon occurs.	Cognitive schemas undergo accommodation and assimilation, reconstructing the existing knowledge structure to accommodate the new paradigm of human-machine collaboration, generating interdisciplinary meta-skills, and achieving an upgrade of the skill structure.
Occupation and psychological consequences	Professional competitiveness is declining, facing significant substitution effects and the risk of salary stagnation, mentally trapped in a cognitive echo chamber of information narrowing, and losing subjectivity.	Productivity significantly improves, obtaining skill premium and work autonomy, achieving cognitive boundary expansion and enhanced self-efficacy, and reaching skill evolution.
Al literacy profile	Exhibits a significant lack of technical understanding regarding algorithmic operations and fails to practice critical evaluation of machine outputs.	Possesses comprehensive Al literacy characterized by strong technical comprehension and the ability to critically assess the reliability of Al-generated content.

## Discussion

This study's integrated framework breaks through the previous single-linear assumption about the impact of occupational exposure to artificial intelligence, providing a dialectical and unified theoretical perspective for understanding individual adaptability in the intelligent era. The academic community has long been trapped in a binary paradox of “technological determinism”: the macroeconomic perspective tends to emphasize the complementary dividend of AI, while the cognitive psychology perspective warns of the ability regression caused by technological dependence. Our model effectively bridges the gap between these two types of literature by introducing metacognitive regulation as a core mediating variable. A critical theoretical advancement in our revised framework is the integration of “Generative AI Literacy” as the behavioral manifestation of metacognitive regulation. Recent scale development studies by Liu et al. (2025) demonstrate that high levels of AI literacy—specifically in dimensions like prompt optimization and critical content evaluation—significantly improve job performance. This effect is fully mediated by creative self-efficacy, confirming that AI literacy provides the necessary cognitive tools for individuals to maintain their “manager” status within the distributed system. Furthermore, the capacity to plan and evaluate human-AI interactions, termed “Collaborative AI Metacognition,” explains significant variance in professional success beyond general cognitive abilities. This framework indicates that occupational exposure to artificial intelligence itself is a neutral environmental input, and the individual's adaptive outcome is not solely determined by the penetration rate of technology, but rather depends on the individual's ability to maintain cognitive sovereignty in a distributed cognitive system. According to the extended theory of mind, when the cognitive process extends to external intelligent tools, the ability to monitor and regulate tool use replaces mere memory or computational ability, becoming a key variable in determining the performance of human-machine systems. Therefore, the core theoretical contribution of this study lies in transforming the skill evolution and cognitive echo chamber from mutually exclusive predictions into two potential branches of the same evolutionary process, clarifying that metacognitive monitoring is the decisive force for individuals to avoid falling into an algorithmic comfort zone and to achieve human-machine collaborative evolution.

Within this collaborative evolution, critical thinking functions as the essential protective barrier against the cognitive silos generated by algorithmic feedback loops. As emphasized by Stefkovics and Gere (2026), the ability to skeptically evaluate and contextualize AI-generated content is crucial for maintaining epistemic sovereignty in an increasingly automated information environment. Critical thinking ensures that individuals do not merely optimize their output based on machine logic but instead integrate AI as a powerful yet fallible partner. This intellectual rigor prevents the erosion of foundational expertise and fosters a dynamic skill set that is resilient to technological substitution. Therefore, the synergy between human agency and critical thinking constitutes the micro-foundation of professional sustainability in the era of artificial intelligence. Further investigation reveals that this framework extends beyond micro level psychological mechanisms to offer a lens for interpreting structural shifts within the macro labor market. Rising wage inequality and occupational differentiation may thus stem from divergent individual cognitive offloading strategies. Individuals with high metacognitive monitoring abilities can adopt strategic offloading, utilizing AI as a scaffold for higher order thinking, thereby securing an advantage in skill premiums and career progression. Conversely, those lacking such regulation are often driven by the principle of ease into opportunistic offloading, resulting in skill degradation and the atrophy of core competencies. This micro level Matthew effect accumulates over time, eventually cementing into structural inequality at the societal level. Therefore, individual aversion or appreciation toward algorithms represents more than a mere emotional response; it serves as a defensive or adaptive psychological reaction to the potential loss of cognitive sovereignty.

**Figure 1:**
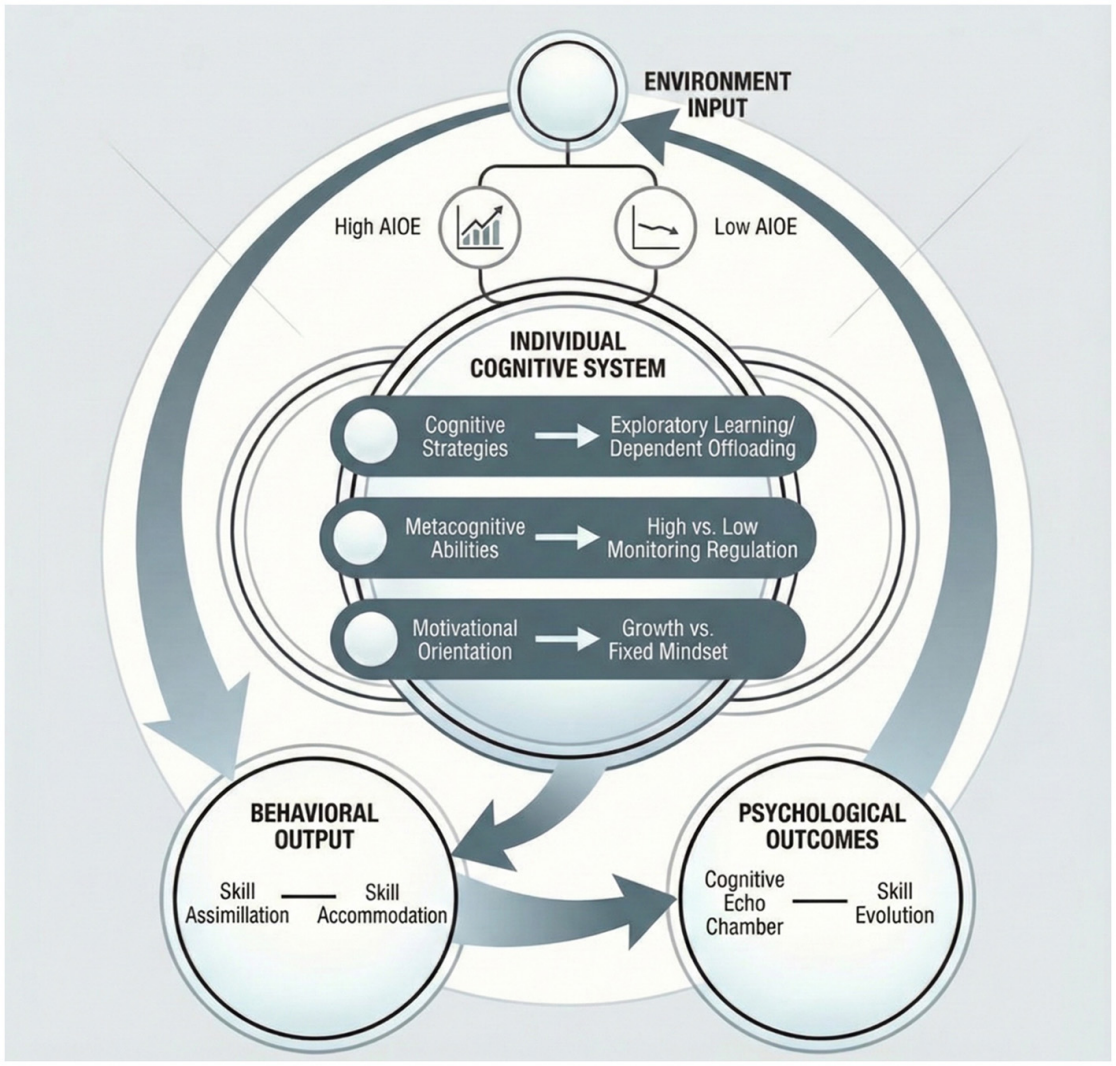
Integrated model of individual cognition and skill evolution in the AIOE environment.

Based on the above theoretical derivation, single technical skill training is no longer sufficient to cope with the deep challenges brought by the exposure of artificial intelligence professions, and we need to build a social-technical ecosystem that supports the development of metacognition. In order to clarify more clearly the guiding significance of the theoretical model for practice at all levels, we constructed Table 2. The integrated model of individual cognition and skill evolution is shown in Figure 1.

**Table 2:** Theoretical challenges and practical path matrix in the AIOE environment.

Level	Key challenges	Theoretical mechanism	Practical path and intervention strategies
Micro individual layer	Cognitive inertia and de-skilling risk	Metacognitive monitoring failure leads to inability to establish dynamic balance between dependence and independence.	Implement metacognitive awareness training, shifting from teaching how to operate AI to cultivating adaptability in when and why to use AI, strengthening belief control and self-efficacy.
Medium-scale organizational layer	Occupational differentiation and organizational rigidity	Distributed cognitive dissonance deprives employees of their judgment and sense of control within the system.	Reconstruct the human-machine loop workflow, design workflows that retain human review rights, establish a culture of prudent algorithm appreciation, and reward behaviors that discover algorithm biases.
Macro-social layer	Digital divide and algorithmic black box trust crisis	Multimodal data complexity and the vacuum of responsibility attribution weaken the regulatory capacity of social systems.	Establish a transparent governance system, promote legislation on algorithmic explainability, lower the threshold for individuals to conduct metacognitive assessments, and build an inclusive technical buffer system.

Furthermore, future research should focus on developing metacognitive measurement tools for the occupational exposure environment of artificial intelligence, and conducting longitudinal studies to empirically test the long-term evolutionary trajectory and critical points of cognitive echo chambers and skill evolution. At the same time, considering the regulatory effect of different cultural backgrounds on algorithmic cognition, cross-cultural comparative studies will also help enhance the universality and explanatory power of this theoretical framework. In summary, only by integrating technological progress with the proactive nature of human cognition can we avoid the trap of cognitive degeneration in the intelligent era and achieve true human-computer symbiosis and skill leapfrogging.

## Limitations

As an opinion article, it must be acknowledged that this framework is currently mainly based on theoretical derivation and literature integration, lacking direct single empirical data support. Although we have integrated empirical studies by Jaccoud (2025) on wage inequality and Baek and Lee (2025) on industrial impact, the model may have simplified the complex non-linear interactions between individual cognitive systems and AIOE. In addition, the specific moderating effects of individual differences (such as prior knowledge reserves) and environmental factors (such as organizational culture) need to be further refined.

## Conclusion

Amid the increasing prevalence of AIOE, it is crucial to recognize that AI technology alone does not dictate whether individuals gravitate toward a cognitive echo chamber or skill evolution. Our integrated framework posits that metacognitive regulation, driven by motivational orientation and executed via cognitive strategies, serves as the critical mechanism distinguishing “passive dependence” from “active adaptation.” Strengthening metacognitive monitoring thus offers a pathway to transform AI from a potential cognitive threat into a driver of evolutionary growth. Consequently, this framework provides a novel theoretical lens on human and AI coevolution, outlining specific avenues for future empirical research and practical intervention.
